# Integrative analysis of lysosome-dependent cell death related molecular subtypes and prognosis prediction in papillary thyroid carcinoma

**DOI:** 10.7150/jca.129191

**Published:** 2026-03-17

**Authors:** Ying Xu, Qiong Wang, Na Zhang, Fugeng He, Xiaochun Mao

**Affiliations:** Zhejiang Cancer Hospital, Hangzhou Institute of Medicine (HIM), Chinese Academy of Sciences, Hangzhou, Zhejiang, 310022, China.

**Keywords:** papillary thyroid carcinoma, lysosome-dependent cell death, prognosis, immune infiltration, LMTK3

## Abstract

**Background:**

Papillary thyroid carcinoma (PTC), the most common thyroid malignancy, shows marked clinical heterogeneity despite generally favorable outcomes. Lysosome-dependent cell death (LDCD), a form of programmed death triggered by lysosomal membrane permeabilization, has emerged as a potential cancer therapy target, but its role in PTC remains unclear.

**Methods:**

Transcriptomic data from public cohorts were analyzed to identify LDCD-related genes (LDCDRG) associated with PTC prognosis. Cox analysis and LASSO regression analyses were performed to construct a prognostic model. Immune landscape, drug sensitivity, and single-cell expression profiles were examined. Functional experiments were conducted *in vitro* to verify the biological effects of the key gene *LMTK3* on PTC cell proliferation, viability, and invasion.

**Results:**

Nineteen LDCDRG were differentially expressed between normal and tumor tissues, defining three molecular subtypes with distinct immune and prognostic profiles. A six-LDCDRG signature (*LMTK3*, *MCM5*, *NXF1*, *TUBB4B*, *LIMCH1* and *APH1B*) effectively stratified patients into high- and low-risk groups with significantly different survival outcomes and acceptable predictive performance. High-risk patients showed reduced immune infiltration and lower predicted immunotherapy-related immune activity. LMTK3, the highest-risk gene, was highly expressed in PTC cells, and its knockdown suppressed proliferation and invasion *in vitro.*

**Conclusions:**

The established six-LDCDRG signature provides an exploratory tool for risk stratification and survival prediction, while *LMTK3* emerges as potential target worthy of further investigation. These findings deepen our understanding of lysosome-dependent cell death in thyroid carcinogenesis and may provide insights into the development of personalized management strategies and novel treatment approaches for high-risk PTC patients.

## Introduction

Papillary thyroid carcinoma (PTC) is the most common type of thyroid cancer, accounting for approximately 80-85% of all thyroid malignancies 1. Although most patients with PTC have a good prognosis through standard treatments such as surgery and radioactive iodine therapy, clinical heterogeneity remains a major challenge 2, 3. Some patients exhibit aggressive tumor behaviors, including extrathyroidal dilation, lymph node metastasis or distant dissemination, resulting in a significantly poor prognosis 4. Therefore, improving risk stratification is crucial for identifying patients with a higher risk 5, 6. Incorporating molecular and clinicopathological features into prognostic models can promote personalized management strategies and optimize treatment decisions 7.

Lysosomes play a key role in the terminal stage of autophagy by mediating the degradation of intracellular components 8. Lysosomal dysfunction leads to autophagy obstruction by damaging endocytic transport or inhibiting substrate degradation 9. Alterations in lysosomal function have been proven to endow tumor cells with enhanced proliferation ability and resistance to treatment 10. Compared to normal cell types, tumor cells rely more on lysosomal activity to support their rapid proliferation and metabolic requirements, making them particularly vulnerable to lysosomal dysfunction 11. Lysosome-dependent cell death (LDCD) is a type of programmed cell death mediated by lysosomal hydrolases. The hallmark of LDCD is lysosomal membrane permeabilization (LMP), which leads to the release of hydrolytic enzymes into the cytosol and subsequent cellular demise 12. Given the significant role of lysosomes in the survival and homeostasis of cancer cells, many small molecule compounds targeting lysosomes have been developed. These drugs can induce LMP or disrupt lysosomal function, thereby selectively killing tumor cells 13. Therefore, further research on the mechanism and therapeutic potential of LDCD is of great significance in the fields of cancer biology and treatment.

Emerging evidence suggests that LDCD may influence the tumor immune microenvironment through multiple mechanisms. Lysosomal membrane permeabilization and lysosome-mediated stress responses can regulate the release of damage-associated molecular patterns (DAMPs), antigen processing, and inflammatory signaling, thereby shaping immune cell recruitment and activation 14-17. In addition, lysosome-associated pathways are closely linked to autophagy, metabolic reprogramming, and immune evasion, all of which are key determinants of tumor-immune interactions 18-21. However, the role of LDCD-related molecular programs in modulating immune infiltration and immune-related phenotypes in PTC remains largely unexplored. Therefore, a systematic investigation of LDCD-associated transcriptional patterns and their relationship with the immune microenvironment may provide novel insights into PTC biology.

In this study, we systematically analyzed differentially expressed LDCD-related genes (LDCDRG) in PTC based on integrated data from public databases and constructed a prognostic model. After validating the model's accuracy in predicting the prognosis of PTC patients, we subsequently conducted analyses including prediction of immune-related characteristics, drug sensitivity analysis, identification of molecular subtype characteristics, pathway enrichment analysis, and immune microenvironment infiltration landscape, to comprehensively evaluate the clinical value of the prognostic model. Single-cell RNA sequencing (scRNA-seq) was used to reveal the cellular subpopulations of PTC and the expression characteristics of LDCD. In addition, LMTK3, the screened gene with the highest risk coefficient, was selected as a representative LDCDRG for *in vitro* experimental analysis. Western blot, Transwell assay, siRNA interference, and CCK-8 assay confirmed that the inhibition of LMTK3 expression significantly suppressed the proliferation and invasion of PTC cells, thereby partially validating the reliability of the bioinformatics findings. Our results suggest a potential role of LDCD in PTC and provide new insights into risk stratification and candidate target for PTC.

## Materials and Methods

### Data Collection

Transcriptomic expression profiles and the corresponding clinical information for papillary thyroid carcinoma (PTC) and normal control (NC) samples were retrieved from The Cancer Genome Atlas (TCGA) database (https://portal.gdc.cancer.gov/). Ensembl gene IDs were annotated and converted to standard gene symbols using custom Perl scripts. Raw count data were normalized and transformed into transcripts per million (TPM) to enable cross-sample comparison, followed by log2(TPM + 1) transformation to reduce the impact of extreme values. Samples with incomplete clinical data or an overall survival (OS) of less than 30 days were excluded to enhance the robustness of subsequent analyses. After quality control, a total of 497 PTC and 59 NC samples were retained for downstream analyses.

### Differential Expression Analysis of Lysosome-Dependent Cell Death-Related Genes

A total of 220 lysosome-dependent cell death-related genes (LDCDRG) were curated based on previously published studies focusing on genes involved in lysosomal function, lysosomal membrane permeabilization, and lysosome-associated cell death pathways (Supplementary Table 1) 22. Differential expression analysis between PTC and NC samples was conducted using the “limma” R package 23, with significance thresholds set at |fold change| ≥ 2 and an adjusted *P* value < 0.05. Heatmaps and volcano plots were generated using the “pheatmap” and “ggplot2” R packages to visualize expression patterns, respectively. To investigate potential protein-protein interactions (PPI), the significantly dysregulated LDCDRG (DE-LDCDRG) were submitted to the STRING database (https://string-db.org/) for network analysis.

### Identification of LDCD Related Molecular Subtypes Landscape

Unsupervised consensus clustering based on DE-LDCDRG expression was conducted using the “ConsensusClusterPlus” R package to identify molecular subtypes of PTC 24. The number of clusters (k) ranged from 2 to 9, using the “k-means” algorithm and Euclidean distance metrics. The analysis was performed with 50 resampling iterations (reps = 50), an item resampling proportion of 0.8 (pItem = 0.8), and a feature resampling proportion of 1 (pFeature = 1). A random seed (123456) was applied to ensure reproducibility. The optimal number of clusters was determined by evaluating the cumulative distribution function (CDF) curve, delta area plot, and consensus matrix heatmap. Principal component analysis (PCA) using the “ggplot2” R package was applied to visualize inter-cluster segregation. Survival differences among subtypes were assessed via Kaplan-Meier (KM) analysis using the “survival” and “survminer” R packages, with significance tested by log-rank tests.

### Pathway Enrichment and Immune Microenvironment Characterization

Gene set variation analysis (GSVA) was conducted using the “GSVA” R package and the KEGG reference gene set (c2.cp.kegg.v7.2.symbols) to identify differentially regulated pathways among molecular subtypes 25. Immune infiltration characterization was quantified using the “estimate” R package, generating immune score, stromal score, ESTIMATE score, and tumor purity for each sample 26. Single-sample gene set enrichment analysis (ssGSEA) was applied to estimate the relative abundance of 23 immune cell types 27. Pearson correlation analysis was used to assess associations between immune infiltration and key variables, and the results were visualized using heatmaps.

### Identification of Differentially Expressed Genes and Construction of the LDCDRG-Based Prognostic Model

Differentially expressed genes (DEGs) between LDCDRG subtypes were identified using the “limma” R package with |fold change| ≥ 1 and adjusted *P* value < 0.05. Functional annotation of DEGs was performed via Gene Ontology (GO) and Kyoto Encyclopedia of Genes and Genomes (KEGG) pathway analyses using the “clusterProfiler” R package. Univariate Cox regression analysis identified survival-associated DEGs, which were further refined via least absolute shrinkage and selection operator (LASSO) regression using the “glmnet” R package to minimize multicollinearity 28. Independent prognostic genes were determined by multivariate Cox regression, and a prognostic risk model was established as follows: LDCDRG score = (2.550 × LMTK3) + (1.948 × MCM5) + (1.803 × NXF1) + (2.263 × TUBB4B) + (0.879 × LIMCH1) + (2.130 × APH1B). Samples were divided into high- and low-risk groups according to the median LDCDRG score. Survival differences were evaluated via Kaplan-Meier analysis. The dataset was randomly split (6:4) into training and validation cohorts using the “caret” R package 29. The predictive performance of the model was assessed using time-dependent ROC curves (1-, 3-, and 5-year) generated by the “timeROC” and “survminer” R packages, with corresponding area under the curve (AUC) values calculated.

### GSEA Evaluation and Nomogram Prediction Model Construction

Gene set enrichment analysis (GSEA) was conducted using the “GSEA” R package with the KEGG reference set (c2.cp.kegg.v7.5.1.symbols.gmt) based on the ranked log2 fold changes of all genes to explore potential biological mechanisms 30. A nomogram integrating clinical variables and the LDCDRG score was constructed using the “rms” R package to predict 1-, 3-, and 5-year OS probabilities. A Sankey diagram generated by the “ggalluvial” R package illustrated the relationships among molecular subtypes, LDCD score, and clinical outcomes.

### Prediction of Immunotherapy Response and Drug Sensitivity

Immunophenoscore (IPS) data were retrieved from The Cancer Immunome Atlas (TCIA) to estimate the potential responsiveness of PTC subtypes to immune checkpoint inhibitors (CTLA-4 and PD-1) (https://tcia.at/home) 31. IPS integrates the expression of MHC molecules, immune checkpoints, effector cells, and immunosuppressive components. Differences in IPS among subtypes were compared to infer potential immunotherapy sensitivity. Drug sensitivity analysis was performed using the “pRRophetic” R package based on the Genomics of Drug Sensitivity in Cancer (GDSC) database (https://www.cancerrxgene.org/) 32. The half-maximal inhibitory concentration (IC50) for various chemotherapeutic and targeted drugs was predicted using a ridge regression model trained on GDSC cell line data. Predicted IC50 distributions were visualized with “ggplot2” R package.

### Single-Cell RNA Sequencing Analysis

The single-cell RNA sequencing (scRNA-seq) dataset GSE184362 was obtained from the Gene Expression Omnibus (GEO) database. scRNA-seq data from five PTC samples (Illumina NovaSeq 6000, 10x Genomics) were analyzed using the “Seurat “ R package (version 4.3.0) 33. Cells with 200-6000 detected genes and mitochondrial gene expression <10% were retained. After log-normalization (“NormalizeData”), 2000 highly variable genes were identified (“FindVariableFeatures”) for subsequent analyses. Data were scaled and subjected to principal component analysis (PCA). The “harmony” algorithm was applied to remove batch effects. Clustering was performed using the “FindNeighbors” and “FindClusters” functions (resolution = 1.2) based on the first 20 PCs, followed by Louvain clustering. Marker genes were identified via the “FindAllMarkers” function (log2FC.threshold = 0.25, adjusted *P* value < 0.05). Cell types were annotated using the “singleR” algorithm referencing “CellMarker” and “PanglaoDB” databases. Two-dimensional visualization was achieved using Uniform Manifold Approximation and Projection (UMAP) and t-distributed Stochastic Neighbor Embedding (t-SNE).

### Cell Culture

Normal human thyroid epithelial cells (Nthy-ori 3-1) and PTC cells (TPC-1) were obtained from the Cell Bank of the Chinese Academy of Sciences (Shanghai, China). Nthy-ori 3-1 cells were cultured in RPMI-1640 medium (Gibco, 11875-093), and TPC-1 cells in high-glucose Dulbecco's Modified Eagle Medium (DMEM; Gibco, 11965-092), both supplemented with 10% fetal bovine serum (FBS; Gibco, 10099-141) and 1% penicillin-streptomycin (Gibco, 15140-122). Cells were maintained at 37°C in a humidified incubator containing 5% CO₂ and subcultured at a 1:3 ratio when reaching ~80% confluence.

### Western Blot Analysis

Protein expression of LMTK3 in Nthy-ori 3-1 and TPC-1 cells was analyzed via Western blotting. Total protein was extracted using RIPA lysis buffer (Beyotime, P0013B) containing protease inhibitors (Beyotime, P1005). Protein concentration was quantified using a BCA kit (Beyotime, P0010). Equal protein amounts (30 μg) were separated on 10% SDS-PAGE and transferred onto PVDF membranes (Millipore, IPVH00010). Membranes were blocked with 5% non-fat milk in TBST for 1 h and incubated overnight at 4°C with anti-LMTK3 (1:1000, Abcam, ab226592) and anti-GAPDH (1:5000, Proteintech, 60004-1-Ig). After washing, HRP-conjugated goat anti-rabbit secondary antibody (1:5000, ZSGB-BIO, ZB-2301) was applied for 1 h at room temperature. Protein bands were visualized using ECL reagent (Thermo Scientific, 32106) and imaged with a Bio-Rad ChemiDoc™ MP system. Band intensities were quantified using ImageJ software, and relative expression was calculated as the LMTK3/GAPDH ratio.

### siRNA Transfection and Efficiency Validation

Small interfering RNA (siRNA) was used to transiently silence LMTK3 in TPC-1 cells. Specific siRNAs targeting LMTK3 (siLMTK3) and negative control siRNA (siNC) were synthesized by GenePharma (Shanghai, China). Cells were seeded into 6-well plates (1×10⁵ cells/well) and transfected with 50 nM siRNA using Lipofectamine™ 3000 (Invitrogen, L3000015) according to the manufacturer's protocol. After 6 h, the medium was replaced with complete DMEM, and cells were incubated for 48 h before collection. Knockdown efficiency was confirmed by Western blot.

### Colony Formation Assay

Following 48 h of siRNA transfection, TPC-1 cells (500 cells/well) were seeded into 6-well plates and cultured for 10-14 days. Colonies were fixed with 4% paraformaldehyde for 10 min and stained with 0.1% crystal violet for 15 min. Colony numbers were counted using ImageJ software.

### Transwell Migration Assay

Cell migration was evaluated using Transwell chambers (8 μm pore size; Corning, 3422). A total of 5×10⁴ cells in 200 μL serum-free medium were added to the upper chamber, while 600 μL complete medium containing 10% FBS was added to the lower chamber. After 24 h incubation, non-migrated cells were removed, and migrated cells were fixed in 4% paraformaldehyde and stained with 0.1% crystal violet. Migrated cells were counted under a microscope in five random fields.

### CCK-8 Cell Viability Assay

Cell viability was assessed using the Cell Counting Kit-8 (CCK-8; Dojindo, CK04). After siRNA transfection for 48 h, TPC-1 cells (2×10³ cells/well) were seeded into 96-well plates. At 0, 24, 48, 72, and 96 h, 10 μL of CCK-8 solution was added to each well and incubated for 2 h. Absorbance was measured at 450 nm using a microplate reader (BioTek, ELx800).

### Statistical Analysis

All statistical analyses were performed using R software (version 4.5.1), Perl, and GraphPad Prism (version 8.0.1). Kaplan-Meier survival curves were compared using log-rank tests. Correlations between variables were assessed using Pearson's correlation coefficient. The Wilcoxon rank-sum test or Student's t-test was used for two-group comparisons, while one-way ANOVA was used for multiple-group comparisons. *P*-values were adjusted for multiple testing using the Benjamini-Hochberg method, and adjusted *P* value < 0.05 was considered statistically significant. All experiments were independently repeated at least three times, and data are expressed as mean ± standard deviation (SD). Statistical significance was indicated as follows: *P < 0.05, **P < 0.01, and ***P < 0.001.

## Results

### Differential Expression Analysis of LDCDRG and Identification of Molecular Subtypes

**Figure 1:**
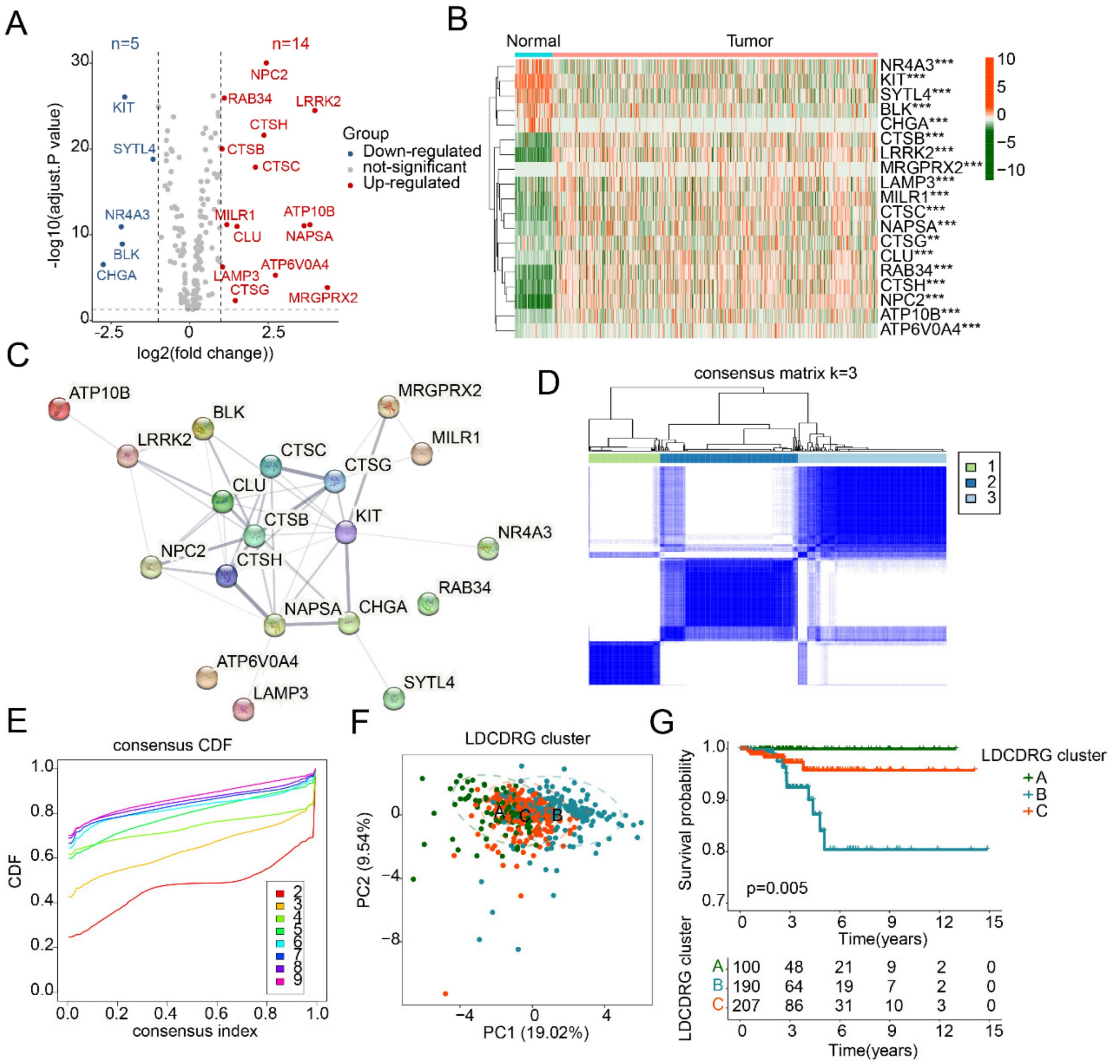
** Differential expression analysis of the LDCDRG signature and characterization of molecular subtype.** (A) Differential expression analysis of the LDCDRG signature between normal and tumor groups, with thresholds set at |fold change| ≥ 2 and adjusted *P* value < 0.05; red plots reprecent upregulate genes, and blue plots reprecent downregulate genes. (B) Heatmap of DE-LDCDRG. (C) Protein-protein interaction network analysis of DE-LDCDRG. (D, E) Consensus clustering analysis for identification of molecular subtype landscape. (F) Principal component analysis of different LDCDRG cluster. (G) Kaplan-Meier survival analysis of clinical outcomes stratified by LDCDRG molecular subtypes.

To investigate the potential regulatory role of the LDCDRG signature in PTC development, a total of 221 LDCDRG-related genes were included in this study, and the differential expression between normal and PTC tissues was analyzed. Using |fold change| ≥ 2 and an adjusted *P* value < 0.05 as the screening criteria, 19 differentially expressed LDCDRG (DE-LDCDRG) were identified, including 5 downregulated and 14 upregulated genes (Figure 1A). Heatmap analysis showed that NR4A3, KIT, SYTL4, BLK, and CHGA were highly expressed in normal tissues, whereas CTSB, LRRK2, MRGPRX2, LAMP3, and MILR1 were markedly upregulated in tumor tissues (Figure 1B). Protein-protein interaction network analysis further revealed potential functional interactions among the 19 DE-LDCDRG (Figure 1C). To systematically characterize the molecular regulatory patterns of LDCDRG in PTC, consensus clustering analysis based on DE-LDCDRG expression profiles was conducted to identify distinct molecular subtypes. According to the consensus clustering results and optimal clustering parameters, samples were classified into three LDCD molecular subtypes: Subtype A (n = 100), Subtype B (n = 190), and Subtype C (n = 207) (Figures 1D, E). PCA patterns demonstrated a clear separation among the three subtypes, further confirming the molecular differences (Figure 1F). Clinical survival analysis indicated significant differences in prognosis across LDCDRG subtypes, with Subtype A showing significantly better outcomes compared to Subtypes B and C (Figure 1G, P = 0.005), suggesting the potential prognostic value of LDCDRG molecular subtypes.

### Immune Microenvironment Characteristics and Immunotherapy Response of LDCDRG Subtypes

**Figure 2:**
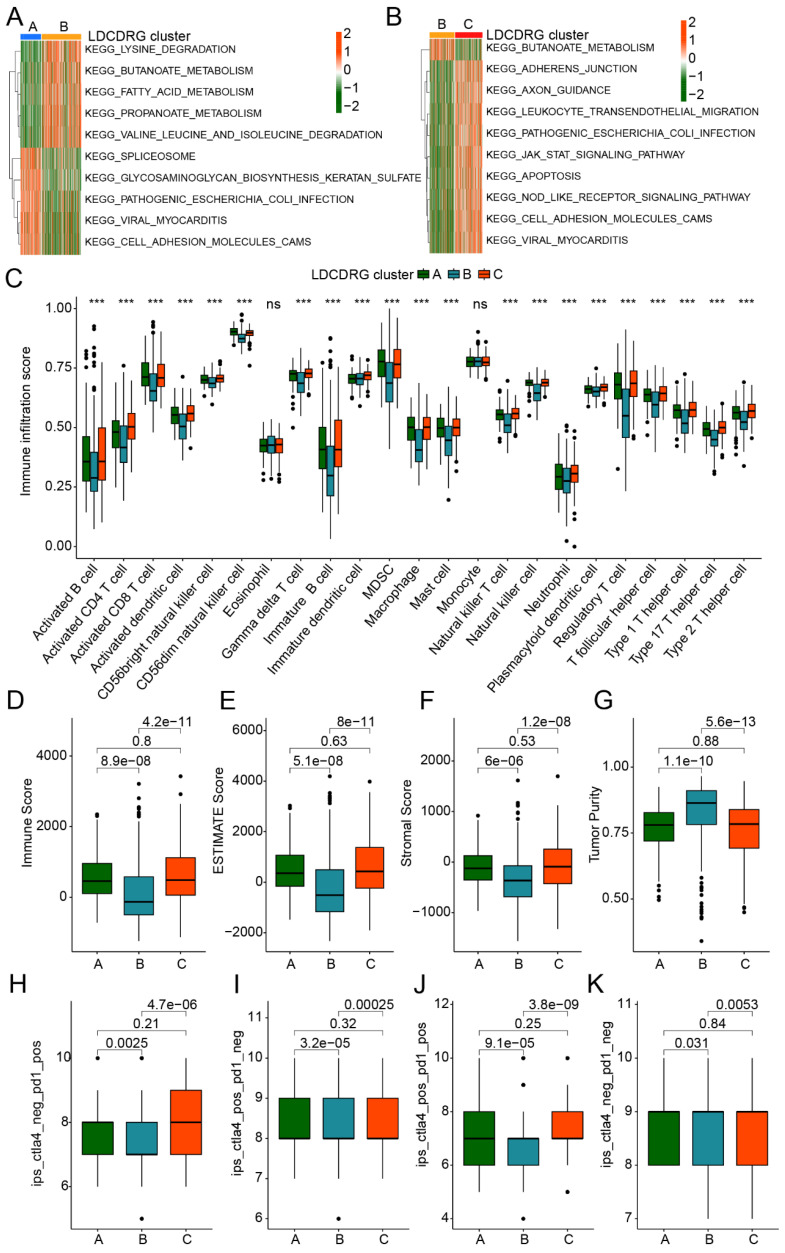
** Immune microenvironment infiltration landscape and immunotherapy response assessment of LDCDRG molecular subtypes.** (A, B) Differential regulation analysis of KEGG signaling pathways among LDCDRG molecular subtypes. (C) Assessment of the infiltration proportions of 23 immune cell types based on the ssGSEA algorithm. (D-G) Evaluation of the immune microenvironment infiltration status. (H-K) Prediction of immunotherapy response for LDCDRG molecular subtypes.

Previous studies have demonstrated that various immune cell populations within the PTC tumor microenvironment play critical roles in immune evasion and prognosis by modulating immune responses. We further assessed immune infiltration differences and potential immunotherapy responses across LDCDRG subtypes. GSVA analysis revealed that, compared with Subtype A, Subtype B showed significant upregulation of metabolism-related KEGG pathways, including lysine degradation, butanoate metabolism, fatty acid metabolism, and propanoate metabolism, while pathways such as spliceosome, glycosaminoglycan biosynthesis, and cell adhesion molecules (CAMs) were significantly downregulated. Compared with Subtype B, Subtype C exhibited significant upregulation of multiple immune-related pathways, including leukocyte transendothelial migration, NOD-like receptor signaling, and CAMs pathways (Figures 2A, B). Based on ssGSEA analysis, the immune infiltration landscape of LDCDRG subtypes was further delineated. Most immune cell populations in Subtype B, including activated B cells, CD4^+^ T cells, CD8^+^ T cells, activated dendritic cells, CD56^bright NK cells, and immature B cells, were significantly reduced, suggesting an immunosuppressive state (Figure 2C). ESTIMATE analysis further confirmed that the clinically worst prognosis subtype B had significantly lower Immune Scores, Stromal Scores, and ESTIMATE Scores, alongside higher tumor purity (Figures 2D-G). IPS analysis indicated that Subtypes A and C had significantly higher IPS than Subtype B, suggesting they may exhibit immune features associated with higher IPS (Figures 2H-K). Collectively, these results reveal substantial differences in immune infiltration and immune-related characteristics among LDCDRG subtypes, indicating that low immune infiltration may contribute to poor prognosis in PTC.

### Comprehensive Prognostic Value of the LDCDRG Scoring System

**Figure 3:**
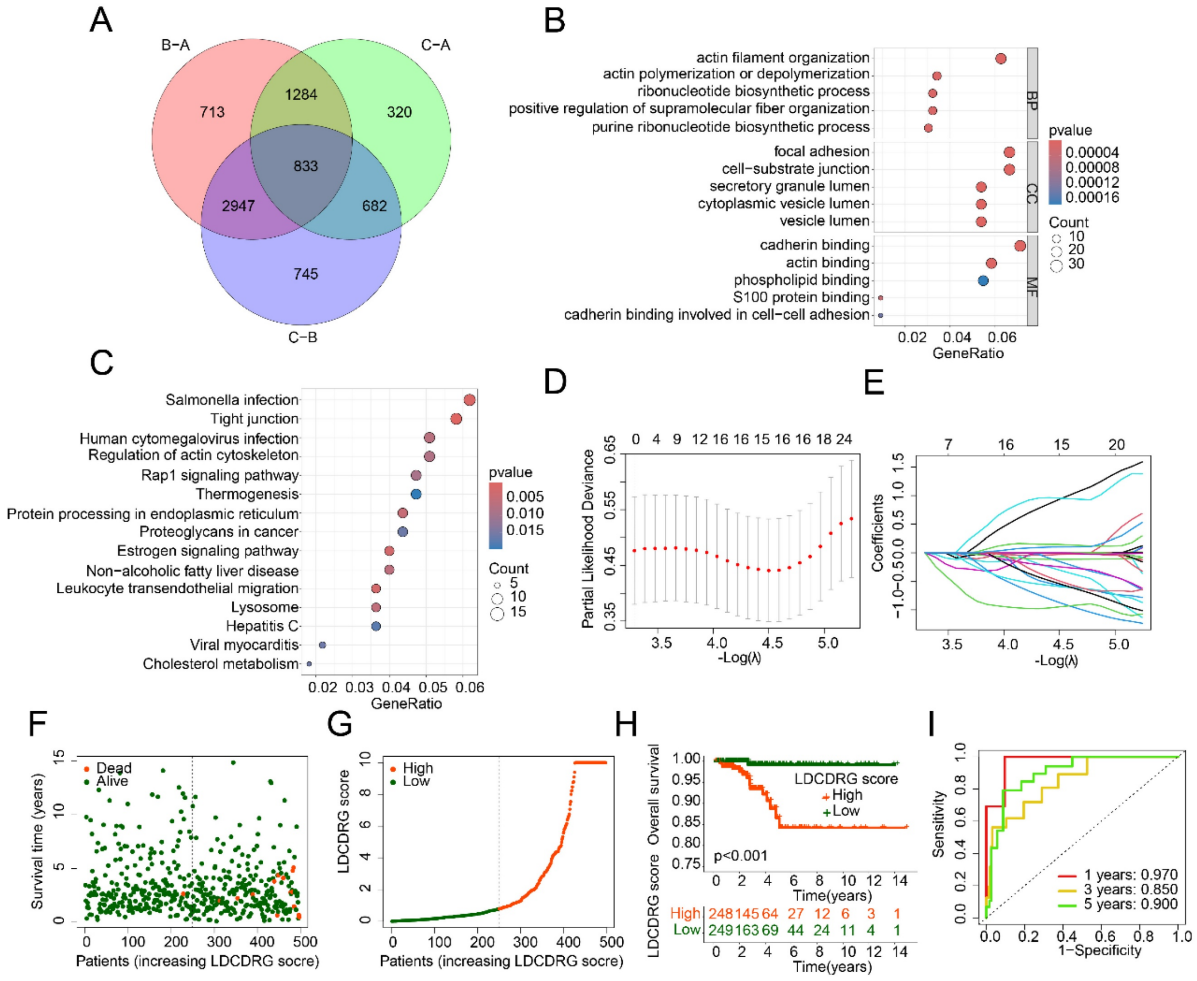
** Differential gene expression analysis among LDCDRG molecular subtypes and construction of the LDCDRG scoring index model.** (A) Differential expression analysis of genes among LDCDRG molecular subtypes. (B, C) GO and KEGG enrichment analyses of differentially expressed genes. (D, E) Feature selection for prognostic variables using LASSO regression analysis. (F, G) Classification of samples based on the LDCDRG scoring index. (H) Kaplan-Meier survival analysis of clinical outcomes stratified by LDCDRG scoring index subgroups. (I) Time-dependent ROC curve analysis.

To explore the mechanisms underlying prognostic differences among LDCDRG subtypes, 833 DEGs were identified between LDCD subtypes (Figure 3A). GO enrichment analysis indicated that these DEGs were mainly involved in actin filament organization, actin polymerization or depolymerization, focal adhesion, and cadherin binding. KEGG pathway analysis suggested involvement in salmonella infection, tight junctions, human cytomegalovirus infection, and regulation of the actin cytoskeleton (Figures 3B, C). Univariate Cox regression analysis identified 86 genes significantly associated with prognosis (Supplementary Figure 1). LASSO regression further refined this list to 15 key features, which were subsequently used to construct the LDCDRG scoring index via multivariate Cox regression (Figure 3D, E). Based on the median score, PTC samples were stratified into high- and low-score groups (Figure 3F, G). Survival analysis revealed that patients in the high LDCDRG score group had significantly worse OS rate compared to those in the low-score group (*P* < 0.001) (Figure 3H). Time-dependent ROC analysis showed that the scoring system predicted 1-, 3-, and 5-year survival with AUCs of 0.970, 0.850, and 0.900, respectively, indicating favorable prognostic performance (Figure 3I).

### Stability and Independence Validation of the LDCDRG Scoring System in PTC Prognosis

**Figure 4:**
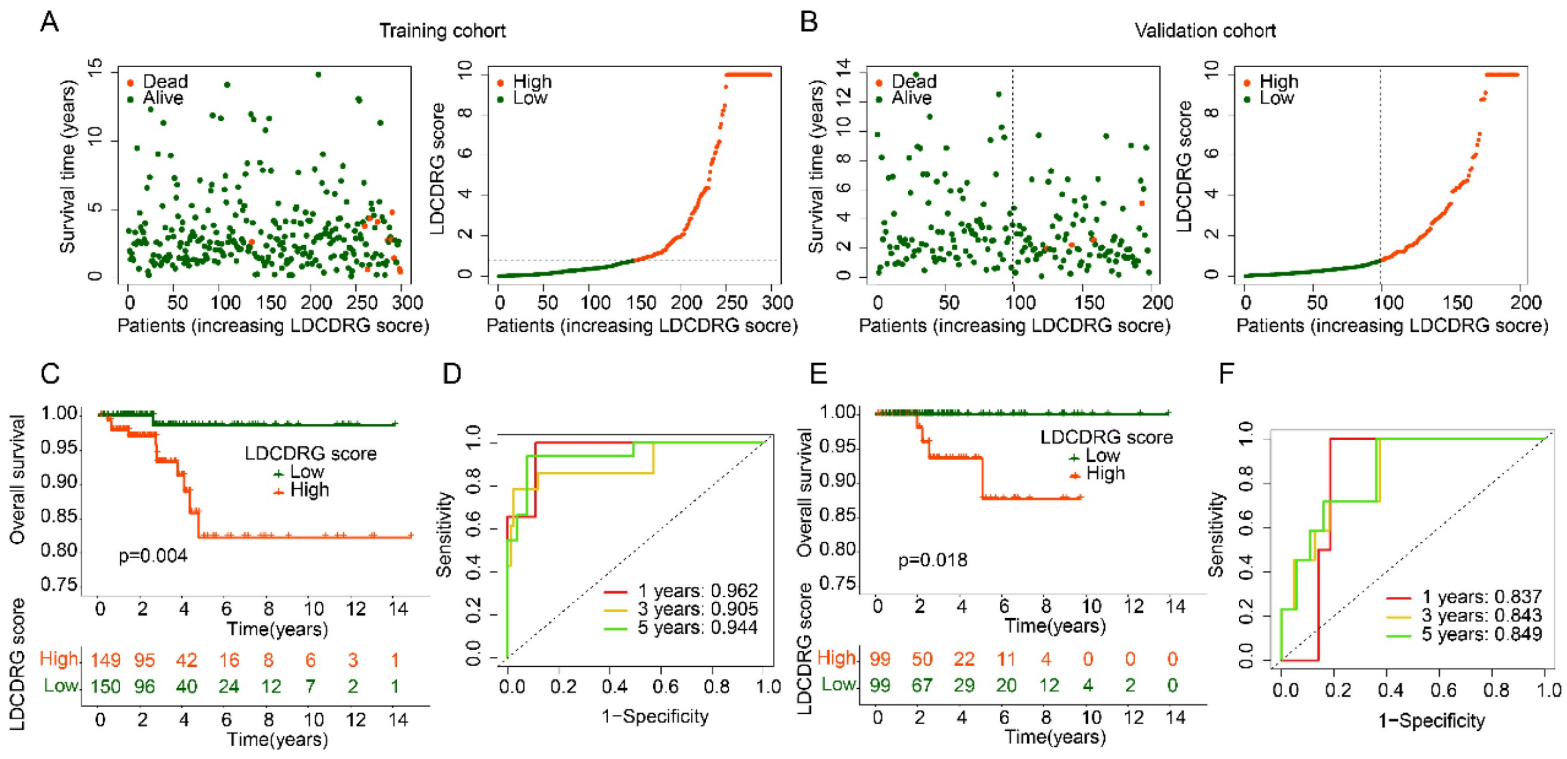
** Consistency and stability analysis of the LDCDRG scoring index.** (A, B) Classification of LDCDRG scoring index subgroups in the training cohort and validation cohort. (C) Kaplan-Meier survival analysis of LDCDRG scoring index subgroups in the training cohort. (D) Time-dependent ROC curve analysis in the training cohort. (E) Kaplan-Meier survival analysis of LDCDRG scoring index subgroups in the validation cohort. (F) Time-dependent ROC curve analysis in the validation cohort.

To assess the stability and independence of the LDCDRG scoring system, PTC samples were randomly divided into a training set and a validation set at a 6:4 ratio. Using the median LDCDRG score, each cohort was separated into high- and low-score groups (Figures 4A, B). Survival analysis demonstrated that low-score groups had significantly higher OS than high-score groups in both training and validation sets, confirming the scoring system's effectiveness in risk stratification (Figures 4C, E). Time-dependent ROC analysis indicated that the scoring system predicted 1-, 3-, and 5-year survival with AUCs of 0.962, 0.905, and 0.944 in the training set and 0.837, 0.843, and 0.849 in the validation set, demonstrating reliable predictive performance across datasets (Figures 4D, F). Overall, the LDCDRG scoring system accurately stratifies PTC patients by risk, providing an exploratory tool for clinical prognosis assessment and potential guidance for individualized therapy.

### Correlation of LDCDRG Scoring Index with Clinicopathological Features

**Figure 5:**
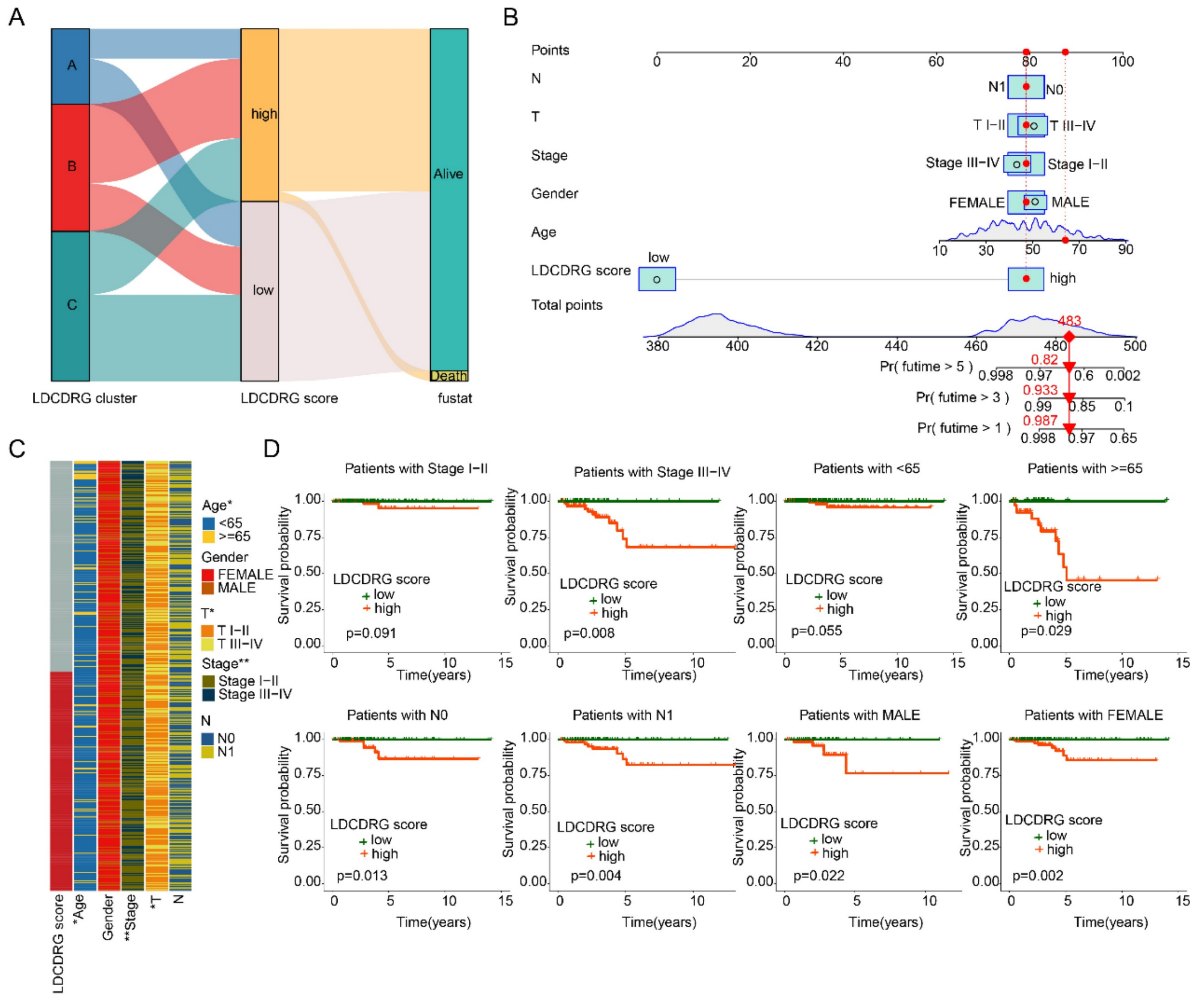
** Construction of the nomogram predictive model and clinical-pathological subgroup analysis.** (A) Sankey diagram illustrating the potential relationships among LDCDRG molecular subtypes, LDCDRG scoring index, and clinical survival outcomes. (B) Construction of a nomogram predictive model based on the LDCDRG scoring index and clinicopathological features. (C) Comparison of LDCDRG scoring index across different clinicopathological variables. (D) Kaplan-Meier survival analysis of LDCDRG scoring index subgroups stratified by different clinicopathological variables.

We further evaluated the prognostic value of the LDCDRG scoring index across different clinicopathological features of PTC samples. Sankey diagram analysis revealed that the poor-prognosis LDCDRG Subtype B was closely associated with higher LDCDRG scores and unfavorable clinical outcomes (Figure 5A). Based on clinical features and the LDCDRG scoring index, a nomogram was constructed to accurately predict 1-, 3-, and 5-year survival probabilities in PTC patients (Figure 5B). Heatmap analysis showed significant differences in LDCDRG scores across age, stage, and N stage (Figure 5C). Subgroup survival analysis demonstrated that in patients stratified by gender (female vs. male), age ≥ 65 years, stage III-IV, and N stage (N0 vs. N1), the low-score group had significantly better survival outcomes compared to the high-score group; however, in stage I-II and age < 65 years subgroups, the survival differences did not reach statistical significance (Figure 5D). These results indicate that the LDCDRG scoring index can effectively stratify risk when combined with clinicopathological features, providing a useful tool for individualized prognostic evaluation in PTC.

### Immune Microenvironment Characteristics of LDCDRG Scoring Subgroups

**Figure 6:**
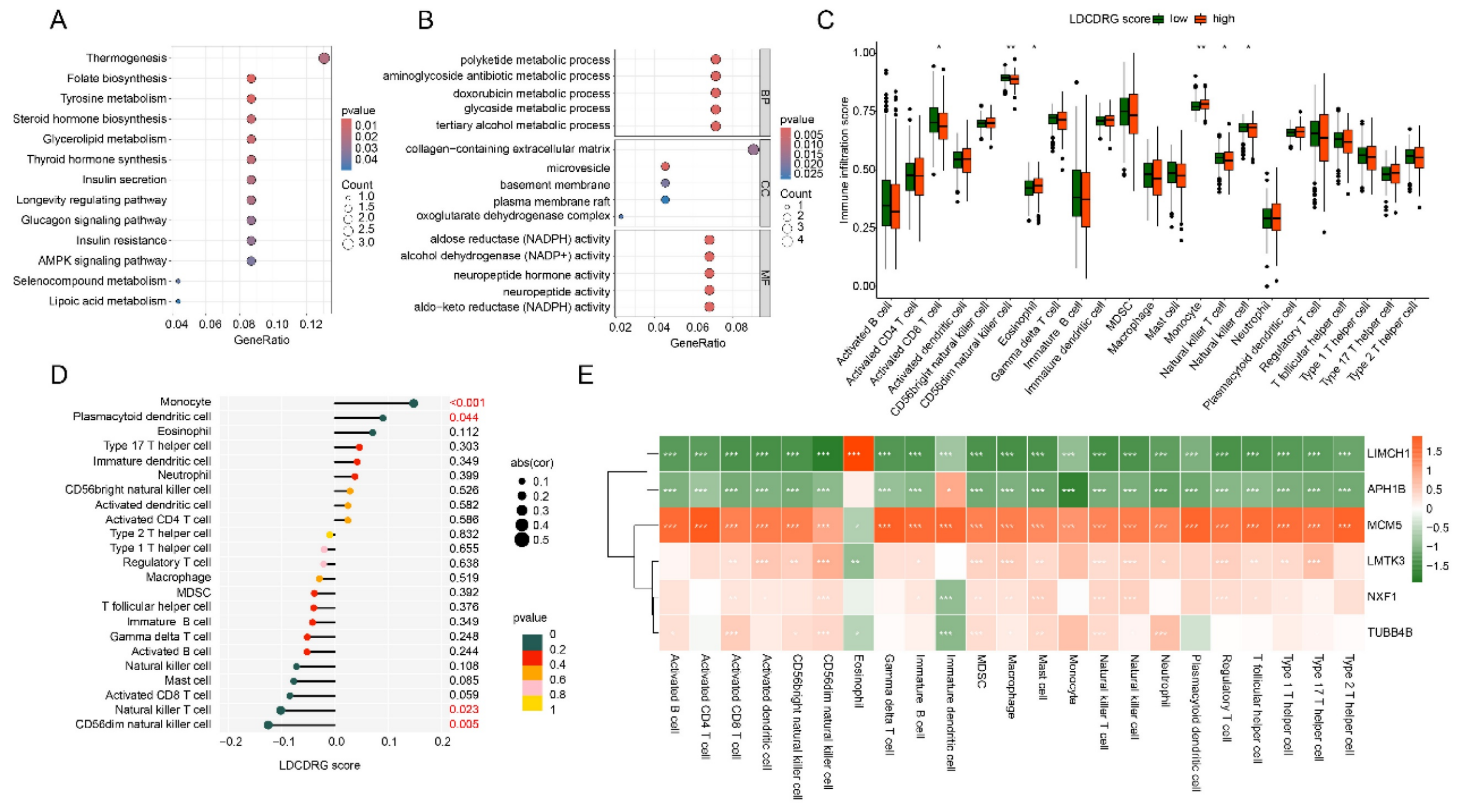
** Immune microenvironment infiltration landscape and correlation analysis of LDCDRG scoring index subgroups.** (A, B) GO and KEGG enrichment analyses of DEGs among LDCDRG scoring index subgroups. (C) Quantitative assessment of the infiltration proportions of 23 immune cell types in LDCDRG scoring index subgroups using the ssGSEA algorithm. (D) Correlation analysis between the LDCDRG scoring index and immune cell infiltration. (E) Correlation analysis between the LDCDRG prognostic signature and 23 immune cell types.

Based on differential expression analysis, DEGs between high- and low-LDCDRG scoring subgroups were identified. KEGG enrichment analysis indicated that these DEGs were primarily involved in steroid hormone biosynthesis, tyrosine metabolism, folate biosynthesis, and thermogenesis pathways. GO functional enrichment revealed involvement in polyketide metabolic processes, aminoglycoside antibiotic metabolic processes, collagen-containing extracellular matrix, and NADPH-dependent aldose reductase activity (Figures 6A, B). ssGSEA analysis was conducted to quantify immune infiltration patterns in LDCDRG scoring subgroups. The low-score group exhibited significantly higher infiltration of CD8^+^ T cells, CD56^dim NK cells, natural killer T cells, and NK cells, whereas eosinophils and monocytes were significantly reduced (Figure 6C). Correlation analysis further demonstrated that the LDCDRG score was significantly negatively correlated with CD56^dim NK cells and natural killer T cells, and positively correlated with monocytes and plasmacytoid dendritic cells (Figure 6D). Additionally, prognosis-related genes LIMCH1 and APH1B showed significant negative correlations with most immune cells, whereas MCM5, LMTK3, NXF1, and TUBB4B were positively correlated (Figure 6E).

### Drug Sensitivity Prediction and Immunotherapy Response of LDCDRG Scoring Subgroups

**Figure 7:**
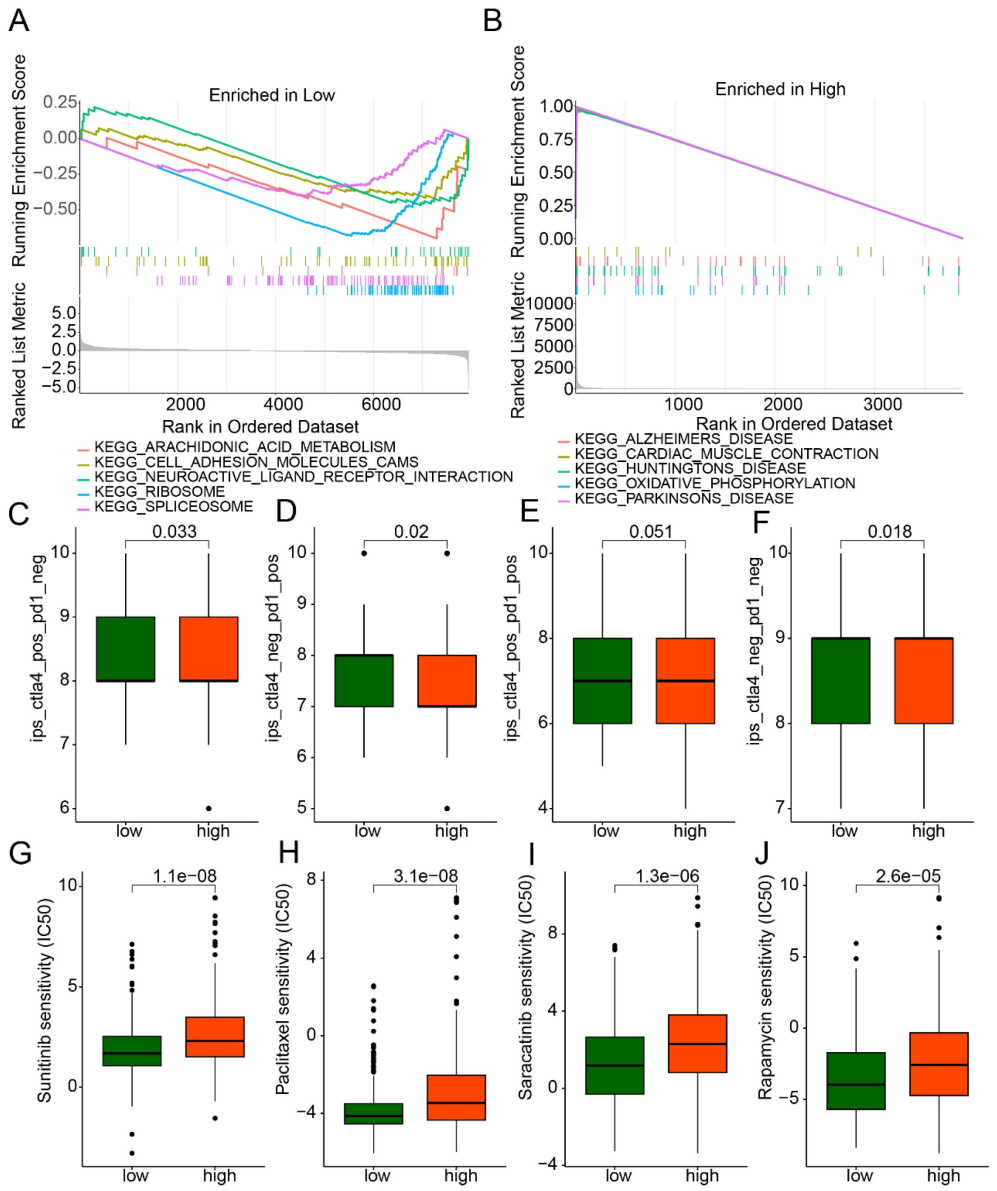
** GSEA analysis and drug sensitivity prediction of LDCDRG scoring index subgroups.** (A, B) Gene set enrichment analysis (GSEA) of LDCDRG scoring index subgroups. (C-F) Assessment of immunotherapy response in LDCDRG scoring index subgroups. (G-J) Prediction of drug sensitivity in LDCDRG scoring index subgroups.

GSEA analysis revealed that in the low-LDCDRG score subgroup, pathways including arachidonic acid metabolism, cell adhesion molecules (CAMs), neuroactive ligand-receptor interaction, ribosome, and spliceosome were significantly upregulated; whereas in the high-score subgroup, oxidative phosphorylation and multiple disease-related pathways were significantly upregulated (Figures 7A, B). IPS analysis indicated that the low-score subgroup exhibited significantly higher IPS compared to the high-score subgroup, suggesting differential immune features (Figures 7C-F). Drug sensitivity analysis further demonstrated that the low-score subgroup had significantly lower IC50 values for Sunitinib, Paclitaxel, Saracatinib, and Rapamycin, indicating potentially higher therapeutic responsiveness to these drugs (Figures 7G-J).

### Single-Cell Sequencing Analysis Reveals Cell Subpopulations and LDCDRG Signature Characterization

**Figure 8:**
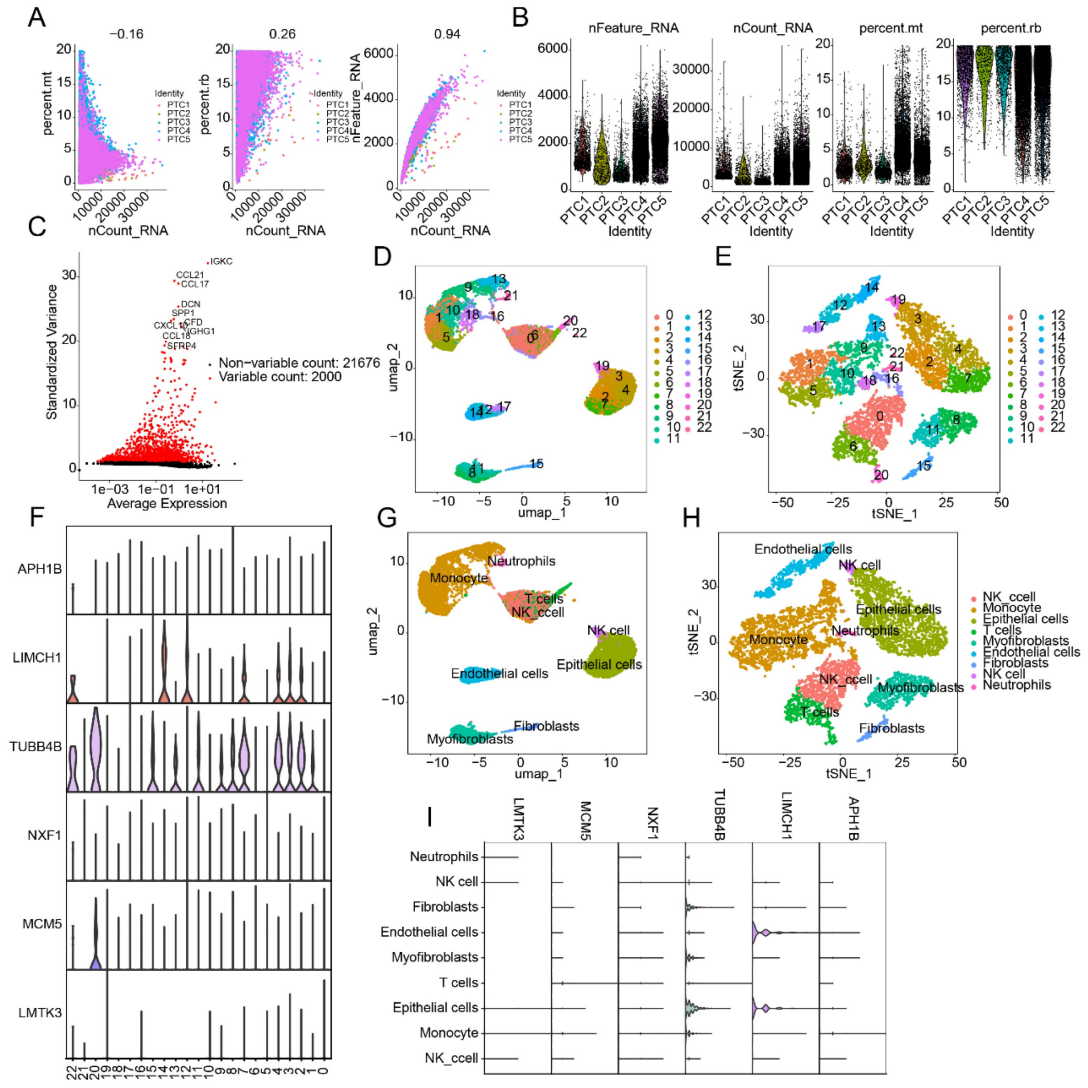
** Single-cell RNA sequencing analysis reveals the distribution features of the LDCDRG prognostic signature.** (A, B) Quality control and normalization preprocessing of scRNA-Seq data from five PTC samples. (C) Identification of the top 2,000 highly variable genes. (D, E) UMAP and t-SNE plots illustrating cell type distributions. (F) Expression patterns of the LDCDRG prognostic signature across different cell types. (G, H) Annotation of cell subpopulations based on the SingleR algorithm. (I) Expression characteristics of the LDCDRG prognostic signature in distinct cell subpopulations.

To further explore cellular heterogeneity in PTC and expression patterns of the LDCDRG prognostic signature, scRNA-seq analysis was performed using the GSE184362 dataset, comprising 5 PTC samples. After quality control and normalization, 2,000 highly variable genes were selected for dimensionality reduction (Figures 8A-C). Based on marker genes, 22 cell types were identified, and their distributions were visualized using UMAP and tSNE plots (Figures 8D, E). Violin plots indicated that six LDCDRG prognostic signature genes were significantly expressed across these 22 cell types (Figure 8F). Using the SingleR annotation algorithm, eight cell subpopulations were accurately identified: NK cells, monocytes, epithelial cells, T cells, myofibroblasts, endothelial cells, fibroblasts, and neutrophils (Figures 8G, H). Violin plots further confirmed that the six LDCDRG signature genes were significantly expressed in all eight subpopulations (Figure 8I), suggesting their broad functional roles within the PTC tumor microenvironment.

### Knockdown of LMTK3 Significantly Inhibits PTC Cell Proliferation and Invasion

**Figure 9:**
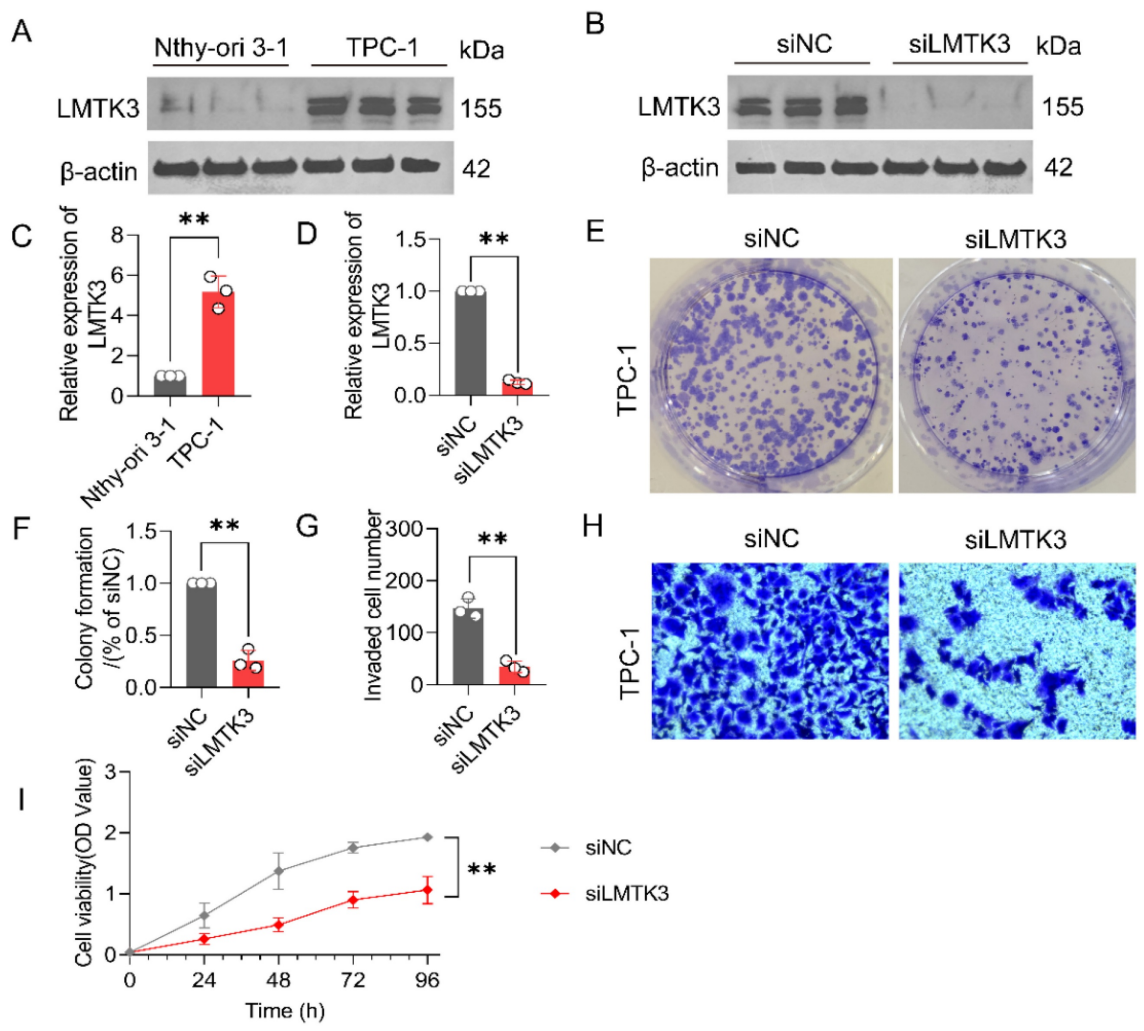
** Knockdown of LMTK3 significantly inhibits the proliferation of PTC cells.** (A) Protein expression levels of LMTK3 in the normal cell line Nthy-ori 3-1 and PTC cell line TPC-1(n=3). (B) Protein analysis of LMTK3 knockdown efficiency. (C) Quantitative analysis of LMTK3 protein levels in Nthy-ori 3-1 and TPC-1 cells (n=3). (D) Comparison of protein expression levels before and after LMTK3 knockdown (n=3). (E, F) Colony formation assays (n=3). (G, H) Assessment of cell invasion capability (n=3). (I) CCK-8 assay measuring cell viability before and after LMTK3 knockdown (n=3). data are expressed as mean ± SD. *P < 0.05, **P < 0.01, and ***P < 0.001.

LMTK3 exhibited the highest risk coefficient in the multivariate Cox regression analysis used to construct the LDCDRG scoring system, suggesting it may serve as a key target associated with poor PTC prognosis. *In vitro* experiments were performed to investigate LMTK3's potential role in PTC cell proliferation and invasion. Western blot analysis showed that LMTK3 protein expression was significantly higher in TPC-1 cell line compared to thyroid Nthy-ori 3-1 cell line (Figures 9A, C). To further verify its function, siRNA-mediated knockdown of LMTK3 was performed in TPC-1 cells. WB confirmed effective LMTK3 knockdown in the siLMTK3 group compared to the siNC control (Figures 9B, D). Functional assays demonstrated that LMTK3 knockdown significantly inhibited TPC-1 cell proliferation in colony formation assays (Figures 9E, F), and Transwell assays indicated a marked reduction in invasive capacity (Figures 9G, H). CCK-8 assays further confirmed that cell viability at 0, 24, 48, 72, and 96 hours was significantly lower in the siLMTK3 group compared to controls (Figure 9I). Taken together, these results indicate that LMTK3 plays a crucial role in regulating PTC cell proliferation and migration, and its knockdown significantly suppresses these oncogenic behaviors, highlighting LMTK3 as a potential therapeutic target in PTC.

## Discussion

Although PTC generally has a favorable prognosis, its significant heterogeneity leads to marked differences among patients in terms of recurrence risk, metastatic potential, and treatment response 34. Our LDCDRG-based risk stratification may provide a framework to support individualized and precise treatment in clinical practice. Given the retrospective design and reliance on public datasets, the present work should be viewed as a hypothesis-generating analysis rather than definitive evidence for clinical application.

Lysosomes play a crucial role in autophagy and are associated with various cancer-related features, including evasion of cell death pathways, immune surveillance escape, and metabolic dysregulation 35. As a result, the role of lysosomes in cancer therapy has gained increasing attention 36. LDCD not only triggers apoptosis and lysosome-dependent cell death pathways but also inhibits cytoprotective autophagy 37. Due to the increased susceptibility of tumor cell lysosomes to LMP, targeting LDCD through LMP-associated signaling in cancer cells may kill tumor cells with minimal impact on normal cells, suggesting a potential for improved safety in treatment 13. Our findings similarly indicate potential relevance of LDCDRG in PTC. Another characteristic of lysosomes is their ability to sequester anticancer drugs in an acidic environment, thereby diminishing drug efficacy. This feature implicates lysosomes in mechanisms of cancer drug resistance 38. Enhancing LMP-mediated LDCD has been shown to offer advantages when combined with conventional chemotherapy in various cancers 39, 40. Whether LDCD-targeted therapies can be combined with standard treatments in PTC remains unexplored and warrants further clinical investigation.

It should be noted that LDCD is not yet a fully standardized or universally accepted category of regulated cell death 41, 42. In the present study, LDCD-related genes were defined in an operational manner, encompassing genes involved in lysosomal structure, function, membrane stability, and lysosome-associated cell death processes, rather than being restricted solely to core executors of LMP. This gene set was curated based on previously published mechanistic and review studies describing the role of lysosomes, LMP, and lysosomal hydrolase release in regulated cell death pathways 43-47. We acknowledge that not all genes included in this set directly mediate LMP or downstream execution of cell death, and some may reflect broader lysosome-related or autophagy-associated processes. Therefore, the LDCD-related gene signature used here should be interpreted as a functional and hypothesis-generating framework, rather than a definitive or exclusive molecular definition of LDCD.

NK cell infiltration in tumors is associated with direct tumor cell killing and immune surveillance and is generally indicative of a favorable prognosis 48. Our immune infiltration analysis showed that lower levels of CD56dim NK cells in the tumor microenvironment correlated with worse outcomes. CD56dim NK cells exhibit greater natural cytotoxicity and produce higher levels of perforin and granzymes 49, 50. The majority of circulating NK cells are of the CD56dim phenotype, which are primarily responsible for rapid elimination of targeted cells 51. Additionally, the CD56dim NK-cell subset expresses higher levels of Ig-like NK receptors and Fc gamma receptor III, enhancing antibody-dependent cell-mediated cytotoxicity (ADCC) 52. These properties underscore the critical role of CD56dim NK cells in both innate and adaptive immunity. Given the important role of NK cells in immune biology 53, future studies may explore PTC risk stratification based on NK cell levels and function.

Our pathway enrichment analysis revealed that fatty acid metabolism may be associated with PTC prognosis stratification. Fatty acid metabolism is essential for thyroid cancer cell energy supply and stress regulation 54. Thyroidectomy leads to significant abnormalities in lipid metabolism among thyroid cancer patients 55. A mouse model study found that enhancing the formation of intracellular free fatty acids and triglycerides to meet energy demands can promote the growth of PTC cells 56. The hypoxic immune microenvironment caused by thyroid cancer progression accelerates tumor development through activation of fatty acid oxidation pathways 57. Fatty acid-binding protein 4 (FABP4), a regulator of lipid metabolism, has also been associated with prognosis in thyroid cancer 58. Fatty acid metabolism has additionally been shown to affect the immune microenvironment of thyroid carcinoma 59. Thus, targeting fatty acid metabolic pathways may have a theoretical basis for treating PTC. Moreover, our results indicated that other metabolic pathways, such as beta-alanine, butanoate, and propanoate metabolism, may also be associated with PTC prognosis, highlighting the important role of metabolic processes in PTC progression.

Our results showed that knockdown of LMTK3 significantly inhibited the proliferation and invasion abilities of PTC cells, suggesting a role for LMTK3 in the process of PTC carcinogenesis. LMTK3 (lemur tyrosine kinase 3) exerts broad functions in cellular signal transduction and membrane trafficking and is overexpressed in various tumors, contributing to tumor progression 60. In thyroid cancer, it has been reported that LMTK3 knockdown can delay cell growth and invasion, which is consistent with our observations 61. LMTK3 was initially identified as a regulator of ERα (estrogen receptor α) in breast cancer 62. Since ERα is overexpressed in thyroid cancer, estrogen antagonists have been proposed as potential therapeutic options 63. Therefore, LMTK3 may serve as a candidate gene for thyroid cancer which warrants further validation. In addition, serum LMTK3 levels are higher in patients with advanced thyroid cancer, and elevated LMTK3 expression has been associated with resistance to multiple chemotherapeutic agents 61, 64-66. Thus, LMTK3 also represents an important indicator for risk stratification in PTC.

In summary, our findings provide new insights into PTC risk stratification and target worthy of further study. However, our study also has limitations. First, although a random 6:4 split was applied to construct the training and validation cohorts, both subsets were derived from the same TCGA dataset. Therefore, the so-called validation represents an internal validation rather than an independent external validation. As a result, the generalizability of the LDCDRG-based prognostic model to other populations and clinical settings remains limited. This limitation is common in high-dimensional bioinformatics studies based on public datasets, and the predictive performance of the model may be overestimated to some extent. Moreover, while we validated the role of LMTK3 *in vitro*, other candidate genes and mechanisms identified from our analyses remain to be experimentally confirmed. In addition, given that immune checkpoint inhibitors are not currently established treatments for PTC, the IPS- and TIDE-based analyses in this study are intended to reflect potential immune characteristics rather than to guide immunotherapeutic decision-making. These results should be considered hypothesis-generating and intended to inform future mechanistic and translational investigations. Future work involving larger prospective cohorts, *in vivo* studies, and clinical trials is warranted to further validate and translate these findings into future clinical validation.

## Supplementary Material

Supplementary figure and table.

## Data Availability

The datasets used and/or analyzed during the current study are available from the corresponding author on reasonable request.
