# Supplementary material for: Integrative analysis of lysosome-dependent cell death related molecular subtypes and prognosis prediction in papillary thyroid carcinoma
Source: J Cancer. 2026 Mar 17;17(3):646–61. doi: 10.7150/jca.129191 (PMC13003606; doi:10.7150/jca.129191)
Supplement: Supplementary file 1 — Supplementary figure and table. [file jcav17p0646s1.pdf]

Supplementary Figure 1. Univariate Cox regression analysis of differentially expressed genes.

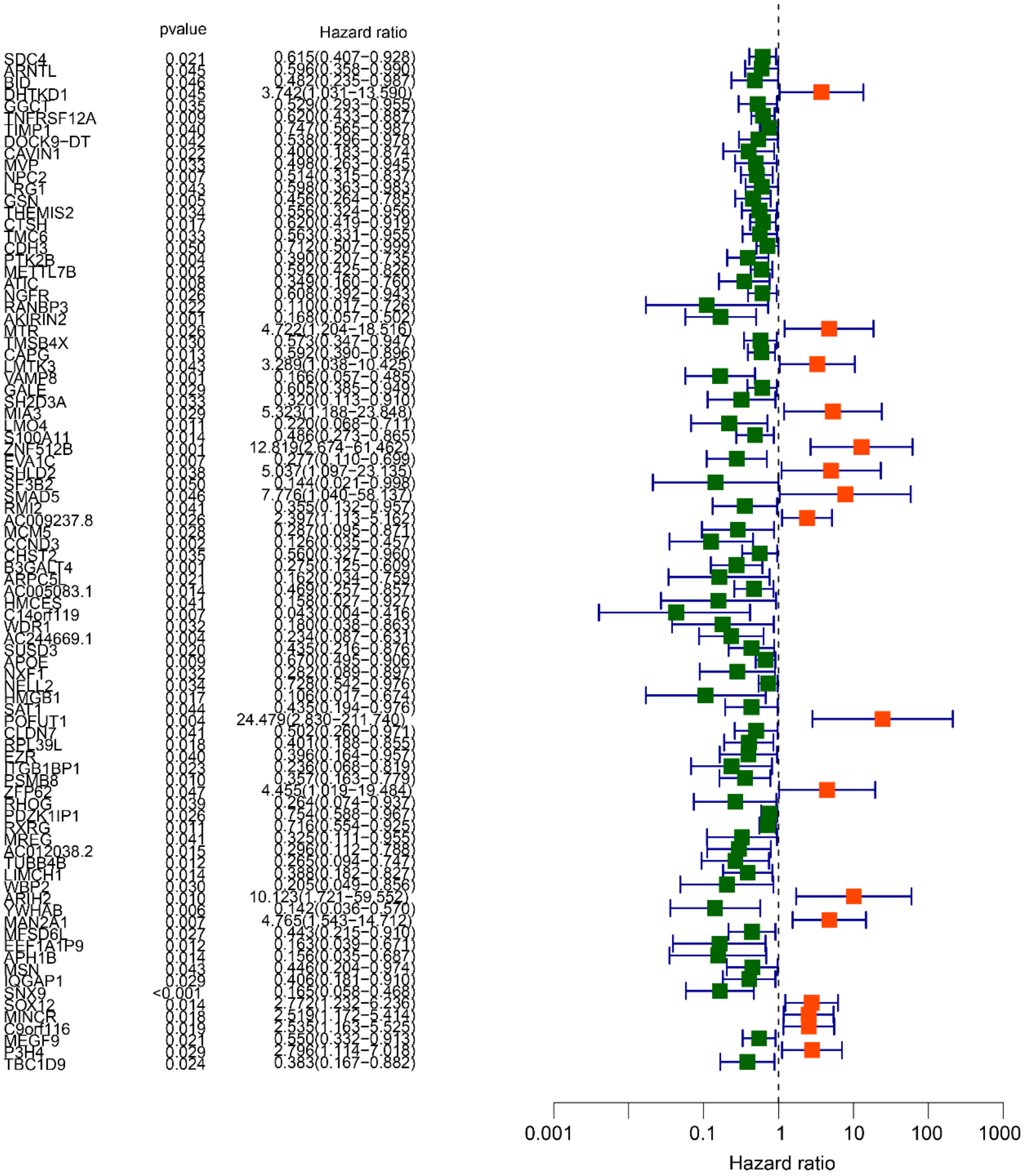

Supplementary Table 1. The gene list of lysosome-dependent cell death–related genes.

ABCA2
ABCB9
ACP2
ACP5
ADGRE2
AGA
AP1B1
AP1G1
AP1M1
AP1M2

AP1S1
AP1S2
AP1S3
AP3B1
AP3B2
AP3D1
AP3M1
AP3M2
AP3S1
AP3S2
AP4B1
AP4E1
AP4M1
AP4S1
ARF1
ARL8B
ARSA
ARSB
ARSG
ASAH1
ATP10B
ATP13A2
ATP6V0A1
ATP6AP1
ATP6V0A2
ATP6V0A4
ATP6V0B
ATP6V0C
ATP6V0D1
ATP6V0D2
ATP6V1H
BLK
BL0C1S1
BL0C1S2
B0RCSS
B0RCS6
BTK
C120rf4
CBL
CD164
CD300A
CD63
CD68

CD84
CHGA
CLN3
CLN5
CLNK
CLTA
CLTB
CLTC
CLTCL1
CLU
CPLX2
CTNS
CTSA
CTSB
CTSC
CTSD
CTSE
CTSF
CTSG
CTSH
CTSK
CTSL
CTSO
CTSS
CTSV
CTSW
CTSZ
DEF8
DNASE2
DNASE2B
ENTPD4
FAM98A
FER
FES
FGR
FLCN
FOXF1
FTH1
FTL
FUCA1
GAA
GAB2
GALC

GALNS
GATA2
GBA
GCC2
GGA1
GGA2
GGA3
GLA
GLB1
GM2A
GNPTAB
GNPTG
GNS
GUSB
HDAC6
HEXA
HEXB
HGS
HGSNAT
HMOX1
HPS6
HSPA8
HYAL1
IDS
IDUA
IGF2R
IL13
IL13RA2
IL4
IL4R
KIF1B
KIT
KXD1
LAMP1
LAMP2
LAMP3
LAMTOR1
LAPTM4A
LAPTM4B
LAPTM5
LAT
LAT2
LGALS9

LGMN
LIPA
LRRK2
LYN
M6PR
MAN2B1
MANBA
MAP1LC3A
MAP6
MCOLN1
MFSD8
MILR1
MRGPRX2
MT3
MYH9
NAGA
NAGLU
NAGPA
NAPSA
NCOA4
NDEL1
NEDD4
NEU1
NPC1
NPC2
NR4A3
PDPK1
PIK3C3
PIK3CD
PIK3CG
PIP4K2A
PIP4K2B
PIP4P1
PLA2G15
PLA2G3
PLEKHM1
PLEKHM2
PPT1
PPT2
PSAP
PSAPL1
PTGDR
PTGDS

RAB34
RAB3A
RAB7A
RAC2
RUBCNL
S100A13
SCARB2
SGSH
SLC11A1
SLC11A2
SLC17A5
SMPD1
SNAP23
SNAPIN
SNX16
SNX4
SORL1
SORT1
SPAG9
SPHK2
SQSTM1
STXBP1
STXBP2
SUMF1
SYK
SYTL4
TCIRG1
TFEB
TMEM106B
TPP1
UNC13D
VAMP7
VAMP8
VPS33A
VPS33B
VPS4A
WASH3P
ZFYVE16
